# Humus gnosis: soil fertility, research and funding in the life of Sir Albert Howard

**DOI:** 10.1017/S0007087424001468

**Published:** 2025-03-13

**Authors:** Tad Brown

**Affiliations:** Department of History and Philosophy of Science, University of Cambridge, UK

## Abstract

Sir Albert Howard helped popularize the idea of translating ‘Eastern’ practice into ‘Western’ science in the field of agriculture. His approach to composting has been foundational to organic farming and counterposed with the field of agricultural chemistry. This depiction of feuding ideologies – organic versus chemical – is based largely on Howard’s opposition to the fragmentation of scientific knowledge and its products, especially artificial fertilizer. One underexplored aspect of Howard’s contest with the agricultural research establishment is the role played by intellectual property. This article contributes to Howard’s historiography by examining three topics related to his life’s work that concern money and patents: (1) the financial support for the Institute of Plant Industry at Indore, (2) an artificial manure patented by employees at Rothamsted Experimental Station and (3) a rival method in British municipal composting. I argue that Howard’s ideological difference with agricultural chemists was not reducible to generating soil fertility with compost. Rather, the feud consisted of a larger debate about innovation, ownership and the societal benefits of scientific research.

In humus you are getting a much more intangible thing. Humus is life, and life is a mystery.

Lieutenant-Colonel F.C. Temple (1941)

Sir Albert Howard (1873–1947) has been celebrated in the annals of science as ‘the father of organic agriculture’.^1^ Although this nomination is a subject of some academic dispute, Howard authored a canonical text of the early organic farming movement, *An Agricultural Testament* (1940), which emphasized the restoration of soil fertility with compost.^2^ According to Howard, ‘the place of organic matter in the soil economy was forgotten’ for a hundred years, a lapse due primarily to the scientific influence of Justus von Liebig, who in the nineteenth century identified inorganic minerals as key to plant growth.^3^ Albert and his wife Gabrielle both studied natural sciences at the University of Cambridge and then relocated to India after the turn of the twentieth century. During more than twenty years of civil service, the pragmatic demands of farming in rural India led the Howards to prioritize the biological wealth of compost above its specific chemical properties. They came to renounce allegiance to their disciplinary training and, more generally, the ‘artificial subdivision of science’.^4^ The Howards’ statements about science, nature and soil health continue to animate debates on farming in India today.^5^

In this paper, I rely on Albert Howard’s archive at St John’s College at Cambridge, including material from his retirement, to consider the role of research funding and ownership in the early history of the organic agriculture movement. Did his disenchantment with science, and with soil chemistry in particular, have anything to do with intellectual property? Drawing patents into the epistemic contests of twentieth-century science raises questions about how the division of knowledge under intellectual property law fed into competing claims for agricultural improvement. This paper contributes to a body of historical works that address the role of industry, patenting and financial capital in scientific knowledge production.^6^ My intervention is not simply that the organic vanguard opposed intellectual property whilst agricultural chemists subscribed to it. Instead, I contend that competing agendas for soil fertility reflected differences in institutional patronage as well as personal beliefs about scientific research and the trajectory of agricultural economies.

Myths about the early organic movement abound. One powerful refrain is that agricultural institutions simply disregarded the Howards’ visionary message. But as historian Philip Conford’s review of periodicals from the 1930s and 1940s has demonstrated, proponents of chemical agriculture did acknowledge the organic school.^7^ Howard’s followers were taken seriously as a threat by the chemical industry, which saw organic proponents as alarmists with an irrational commitment to dung. Composting was a topic of open debate in professional forums. All parties recognized its value to some extent. In fact, Conford showed that the *Fertiliser, Feeding Stuffs and Farm Supplies Journal* hired a chemist for editorial work to fact-check submissions that endorsed compost. The contribution of artificial fertilizers to the First World War created lasting support for the chemical industry and its model of agricultural intensification.^8^ The technology attained a wartime momentum that carried throughout the twentieth century.^9^

Other recent scholarship has sought to dispel myths about the Howards, particularly their antipathy towards chemical fertilizer. By 1924, when the couple founded the Indore Institute of Plant Industry in central India, they had yet to fully commit to the gospel of compost. Historian Gregory Barton has reviewed the Howards’ stance on artificial fertilizers to find that their initial dislike was not based on any perception of unnaturalness.^10^ Rather, the Howards rejected chemical inputs at first due to its lack of affordability for Indian ryots (peasants). The foregrounding of economics is significant for understanding the Howards’ expansive views on compost. Advocates of organic agriculture, like the Howards, were concerned with the effects of artificial chemical fertilizer on both soil health and rural lifeways.

Taking guidance from reports on the ‘Far East’ (China, Korea, Japan), where field practices had sustained populous villages on small-acreage plots for centuries, the Howards’ agrarian philosophy manifested as a total commitment to soil fertility via decomposed organic matter, also known as humus.^11^ According to sociologist Thomas Gieryn, the ‘Howards’ growing conviction that the route to improved cotton growing in India [went] through Chinese farmers rather than through Cambridge laboratories’ was not a retreat from scientific authority but a rural reappraisal of its principles.^12^ One result of their work in India was the Indore process, a composting method for which Sir Albert Howard (knighted 1934) became famous through published works and public lectures. In his own words, *An Agricultural Testament* was a ‘continuation of an earlier book in which the Indore Process for maintaining soil fertility by the manufacture of humus from vegetable and animal wastes was described’.^13^ The methods of manufacture had not changed substantially in this newer text, but Howard’s explanation had.

Historian of science Merlin Sheldrake has discussed the importance in *An Agricultural Testament* of fungi, organisms absent from Howard’s earlier humus theory. Sheldrake argued that the book’s emphasis on mycorrhiza, fungal filaments that free soil nutrients for plant uptake, offered a point of authorial self-reflection. In this view, Albert Howard was ‘closely mirrored in his portrayal of mycorrhizal fungi’ by acting as an intermediary between ‘East’ and ‘West’.^14^ By comparison, historian Sanjukta Ghosh has stated that the Howards mostly deserve credit for recognizing indigenous practices of soil husbandry in India. Through an ‘adaptive empiricism’, the Howards learned to value ryots’ farming methods, an encounter that was instructive yet, as Ghosh clarified, limited by the colonial context in which it occurred.^15^

Many agricultural scientists considered Albert Howard an extremist during his lifetime. Chemical inputs and mechanization had achieved greater output per worker, and for so-called progressive farmers this metric trumped any holistic assertions.^16^ One group, in particular, challenged the properties ascribed to humus by the Howards. Researchers at the Rothamsted Experimental Station in Harpenden, England, were working to advance the field of soil chemistry. In their view, sufficient amounts of nitrogen, phosphorus and potassium helped plants grow, regardless of source. Rothamsted has come to embody a competing school of scientific thought in the Howards’ historiography – the chemical model of agricultural intensification versus the organic. By construing ‘Liebig and his epigones at Rothamsted’ as part of a larger cultural cartography, Gieryn ventured furthest in his analysis to argue that soil science was indispensable for institutional claims to being modern.^17^ Gieryn also noted in passing that employees of the Rothamsted Experimental Station held a utility patent on the manufacture of a commercial fertilizer.^18^ This detail suggests that ownership may have figured in the philosophical discord between Indore and Rothamsted.

Rival statements about compost demonstrate how scientists in the first half of the twentieth century engaged with intellectual property to manipulate the contours of knowledge. In this paper, my inquiry into the Howards’ endorsement of humus proceeds through three parts. First, I consider the financial support of the Indore Institute of Plant Industry. Indore holds interest because the Howards’ initial proposal for the institute stalled from insufficient funding. An industrial tax on Indian cotton eventually enabled its establishment. Next, I turn to the artificial manure patented by employees at Rothamsted, asking whether inventive steps for making the commercial fertilizer interfered with the Howards’ composting claims. The final section addresses the global vision of Sir Albert Howard for the Indore process. During retirement, he declined an offer to join the board of a private municipal composting company. Howard took issue with the efficacy and novelty of the founder’s patented composting method, and he expressed concern that personal affiliation with the British company could compromise the integrity of the Indore process. At the same time, the Indore process faced opposition from institutional rivals and individuals who held patents. These episodes demonstrate how ownership fed into debates about soil fertility and claims to epistemic authority in agricultural science.

## The Indore process

Albert Howard graduated from Cambridge with a diploma of agriculture, and in 1899 he took a post in the West Indies working for the Imperial Department of Agriculture.^19^ Afterwards, he returned to England and joined the staff at Wye College from 1903 to 1905. A colleague later wrote that Howard ‘was not, however, entirely happy at Wye’.^20^ It was during this time that Albert courted his soon-to-be wife, Gabrielle L.C. Matthaei, who stayed in Cambridge following her graduation and held an appointment in chemistry investigating plant transpiration.^21^ The couple got married in 1905. That year, in a letter to Albert, Gabrielle highlighted the functional complexity of crop biology, asserting that ‘the plant knows no division of science’.^22^ This perspective would become a thesis for their life’s work together overseas. ‘The beginning is always difficult’, Albert wrote to his mother-in-law in April 1905 en route to India, having been hired as an imperial economic botanist.^23^ By the following month he was at Pusa, then located in the Bengal Presidency.

**Figure 1:**
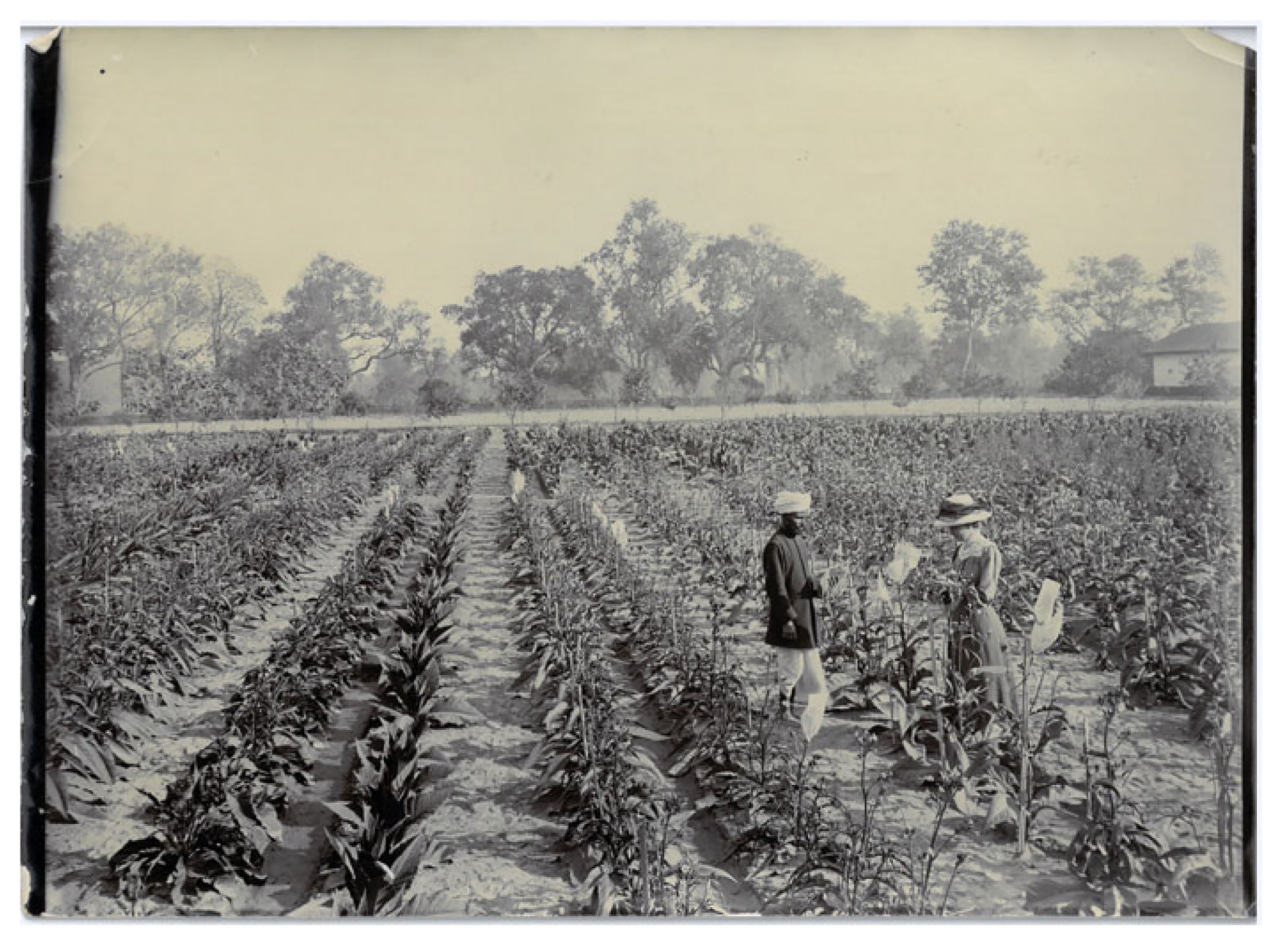
Gabrielle Howard making observations in a field plot alongside a co-worker, whose name is not recorded in the archive. St John’s College Library, the papers of Sir Albert Howard, HowardA/A/5/5. By permission of the Master and Fellows of St John’s College, Cambridge.

The Agricultural Research Institute of Pusa had recently been established as part of the all-India Imperial Department of Agriculture, with the Botanical Section under Howard being the last section organized.^24^ Gabrielle joined Albert as a volunteer later that year. She officially became Albert’s personal assistant in 1910 and was soon thereafter promoted to second imperial economic botanist, an honour unheard of for women in India at the time (Figure 1).^25^ The Howards devoted over eighteen years at Pusa to the classification and breeding of wheat, as well as experimentation with green manures, yet the location left something to be desired.^26^ Not only was their social life in Pusa largely restricted to the experiment station, but seasonal waterlogging limited field studies. The Howards decided to seek a second station. In 1910, they took a trip to Kashmir.^27^ Two years later, they acquired land for a Fruit Experiment Station at Quetta in the high desert of Baluchistan, south-west of Kashmir.^28^

Agricultural items comprised the majority of India’s export trade at over 85 per cent of its total value.^29^ Cotton topped the list. Tired of the bureaucracy and departmentalization of agricultural research, the Howards quit Quetta in 1919 to realize their vision for organizing research around the whole plant, this time through cotton. More than an opportunity to improve exports of long staple cotton, the Howards were attracted by the administrative potential of institutional change. The construction of the Institute of Plant Industry at Indore was delayed, however, from 1919 to 1924. ‘Indeed, had not the Central Cotton Committee stepped in to vote two lacs of rupees for capital expenditure and one lac thereafter for recurring annual costs’, Albert confessed, ‘the proposed venture would never have materialized’.^30^ Funding for the Indore Institute, with its significance in the history of organic agriculture, was thus made possible by an industrial tax on export fibre for the British textile industry.^31^ The Darbar of Indore provided a ninety-nine-year lease to house the institute, with financial support coming from the Indian Central Cotton Committee, eight states of Central India, and Rajputana. These patrons retained seats on the board when the institute incorporated in 1928 and funded activities with paid subscriptions.^32^ Research at Indore served its sponsors’ interests.

Unlike the Institute at Pusa, which had been modelled on the Rothamsted Experimental Station in England, the physical grounds of the Indore Institute were designed to reflect the Howards’ own agrarian philosophy.^33^ The ‘general principle underlying their administration [at Indore] was that the crop was to be treated as a unit and studied in relation to the field and the village; there was to be no division of the scientific work into departments’, a peer recalled.^34^ The Howards designed the institute’s layout according to this principle, refusing to partition activities spatially. They wanted ryots to visit Indore and transform local economic practices through the farm philosophy of ‘a biological whole’.^35^ The most telling detail of the site was its placement of compost piles directly beside the cowshed for ready access to fresh dung and urine-soaked bedding. Colonial officials in India had long complained about ryots burning dried cow dung as cooking fuel, viewing the practice as wasteful.^36^ Based on his observations, Albert Howard felt that one-quarter of available cowpats could be put towards composting to revolutionize soil fertility in India.^37^ Through humus all things seemed possible.

The basic design of the Indore process was as follows: compost pits were dug in pairs, each thirty feet long with sloping sides. Into these went various sources of organic matter – roots, weeds, green manures, night soil, cow dung, livestock bedding, algae and so on. A key to the Indore process was providing the correct ratio of carbon to nitrogen for full decomposition. It was a two-step process to convert crop residues into available plant nutrients and sustain a positive soil nitrogen balance.^38^ When done correctly, the woody material was crushed and green matter withered before being layered, watered and turned three times, resulting in a final product, ready for immediate use by plants in ninety days.

The Indore Institute of Plant Industry was more than a demonstration site. It was also a diploma-granting institution, and the Howards recruited help to assist with its demanding list of responsibilities. One staff member, Yeshwant D. Wad, who joined the Indore Institute as a chemical assistant in 1926, is of particular historical interest.^39^ Wad held a master’s degree from the agricultural college in Coimbatore, where his studies and research on cotton biochemistry were funded by a scholarship from the Indian Central Cotton Committee.^40^ His subsequent research at Indore covered the influence of manure and soil texture on cotton yields, as well as a study on cotton disease resistance.^41^ Consistent with the Howards’ overall philosophy, Wad’s findings highlighted the influence of soil conditions on crop response to plant pathogens.

**Figure 2:**
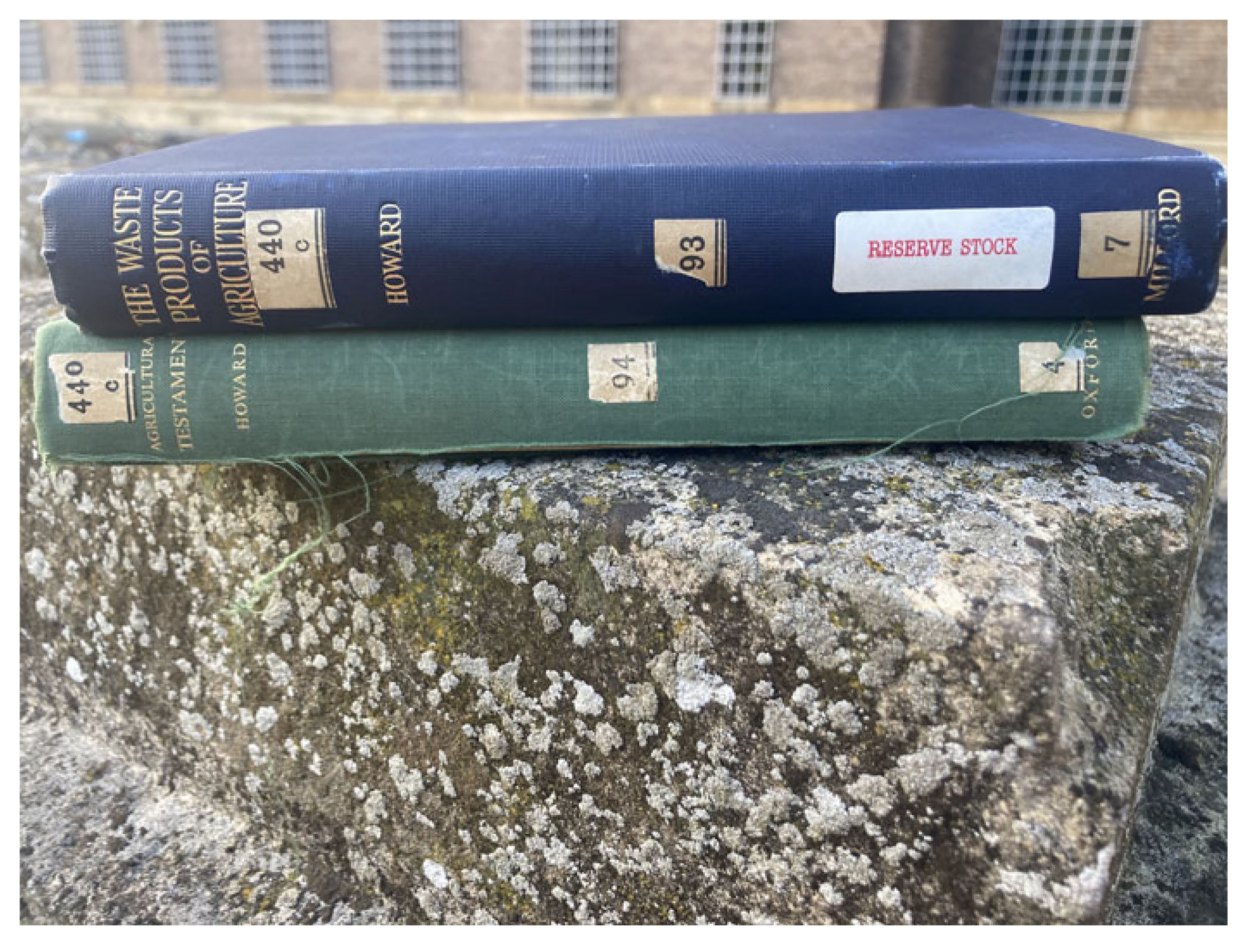
Books from Cambridge University Library. Note that Wad does not appear as an author with Howard on the spine of *The Waste Products of Agriculture*. Photograph by the author.

Wad was co-author with Albert Howard on *The Waste Products of Agriculture* (1931), the book introducing the Indore process. As first author, Howard claimed to have drawn inspiration for the Indore process by studying agricultural history. The groundbreaking text juxtaposed peasants’ conservative treatment of waste in the ‘Far East’ with capital exploits of extensive agriculture in ‘the West’, where imports of Peruvian guano had offset soil exhaustion throughout the nineteenth century.^42^ According to Albert’s second wife, Wad was tasked with analysing ‘the chemical side’ of the Indore process.^43^ His rigorous statistical approach contrasts with the popular image of the methods used at Indore. In fact, the chemist’s contribution has been commonly overlooked, due in part to citations that omit his name (Figure 2).^44^

Wad was chief chemical assistant near the time of Gabrielle’s untimely death in 1930.^45^ He had enjoyed the unique experience of observing the Howards’ daily activities, and he praised their cooperative spirit. ‘Wherever they moved, all was life’, Wad recalled.^46^ The people, plants and livestock at Indore apparently affirmed its curriculum. Like the Howards, Wad viewed the restorative effects of soil fertility as a creed applicable for all societies, especially those ‘destined to halt its present headlong race toward destruction’.^47^ Scientists generally agreed that the physical structure of humus imparted qualities conducive to plant health. They disagreed, however, to what extent agriculture should depend on humus and the vital life lessons it held.^48^

## The ADCO patent

The founding of the Rothamsted Experimental Station shared little in common with the institute in Indore.^49^ It was established in Harpenden, England, with trust money from the sale of Lawes’ Chemical Manure Company by Sir John Bennet Lawes. Historians tend to connect the work at Rothamsted to the legacy of Justus von Liebig.^50^ As contemporaries, Lawes publicly disagreed with Liebig about aspects of mineral theory, particularly Liebig’s idea that sufficient supplies of atmospheric nitrogen fell with rain.^51^ This disagreement played a role in Rothamsted’s early chemical research on soil fertility and industrial developments by Lawes.

**Figure 3:**
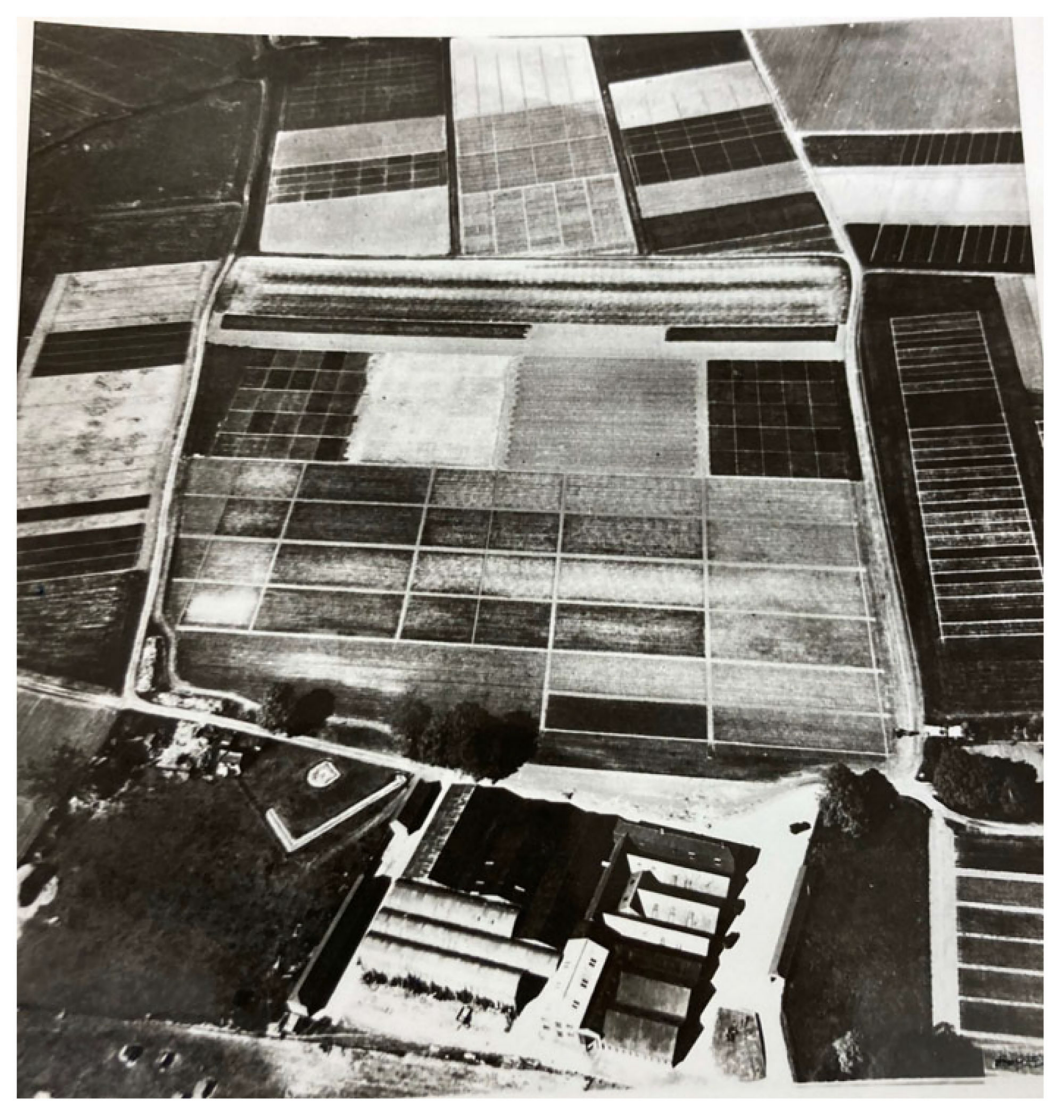
Aerial view of the grounds at Rothamsted Experimental Station. Credit: Museum of English Rural Life, University of Reading, FR HERT 11/6/6, © Rothamsted Research.

Lawes started his Chemical Manure Company after securing a patent in 1842 for the manufacture of the fertilizer known as superphosphate.^52^ What Lawes claimed in his patent was the use of sulphuric acid for obtaining superphosphate ‘for purposes of manure’ from the treatment of charred bones, apatite, guano and mineral phosphate.^53^ Previous publications had already described these chemical reactions or, like Liebig, deduced the importance of minerals from manurial trials. Yet it was experiments by Lawes that revealed the immediate effects of dissolved bone on crop yields.^54^ Competitors in the fertilizer trade led an open resistance against the validity of Lawes’s patent, or, as one attorney expressed, ‘the extraordinary consequence that a person may manufacture superphosphate of lime for one particular purpose but cannot manufacture and use it for agriculture’.^55^ The legal battle caused Lawes to disclaim ownership over the treatment of bone ash and its combination with alkalis, but he retained rights to the use of mineral phosphate. This was enough to secure control of the British trade in phosphatic fertilizers by the 1860s.^56^ After selling the chemical manure company years later, Lawes established a trust to ensure the continuation of Rothamsted’s research, which had done so much for ‘the manufacture of the fertilizer bearing his name’.^57^ The experimental station fulfilled this wish (Figure 3).

Lawes’s patented chemical manure capitalized on major changes in English agriculture. Historically, market gardeners and farmers on the outskirts of London replenished soil fertility with night soil and horse dung, but ‘the chemists and the guano merchants’ dealt an end to the urban–rural manure trade in the 1850s.^58^ Victorian society adopted a new sensibility about farm life to accompany the reconfiguration of agricultural spaces, at once notable in art, legislation and the professional discourse of scientific management.^59^ The value of straw became increasingly unclear as machines replaced livestock on the farm.^60^ In time, two employees at Rothamsted found a way to bypass herbivores with a patented method for turning straw into a substitute for farmyard manure.

Before his work in Harpenden, Eric Richards led purification experiments for the Royal Commission on Sewage Disposal. He then investigated how to deal with the dwindling manure supply for market gardeners in London. Funds for the study – from Rupert Guinness, 2nd Earl of Iveagh – went to the Rothamsted Experimental Station, which hired Richards in 1913.^61^ Henry Hutchinson was on staff as a bacteriologist when Richards joined the station. Both of their studies fed into the subsequent patent. Whereas Richards published on nitrogen fixation during aerobic fermentation, Hutchinson’s research included the influence of plant residues on nitrate loss and the decay of cellulose by cultured bacteria.^62^ Viewed in totality, these ‘epigones of Liebig’ devoted substantial effort to understanding the biological and chemical properties of decomposition.

Richards and Hutchinson filed a patent application to the United States Patent Office in August 1921 for the manufacture of nitrogenous fertilizers. In it, they claimed an invention for recovering nitrogen from soluble solutions. The most basic design, as illustrated in the patent, involved a tank and straw. During aerobic fermentation of waste liquids from sewage works, nitrogen was fixed in the bodies and excreta of microorganisms if kept in contact with a carbonaceous material.^63^ Saturating straw with a diluted sewage solution resulted in an ‘organic agglomerate containing available nitrogen’ similar to farmyard manure.^64^ The patent was granted in 1923.

The following year, these same scientists secured a patent in Great Britain for their invention.^65^ This time, the application was filed with the Agricultural Development Company Ltd (ADCO). ADCO was ‘a non-profit-making syndicate’ organized by Rupert Guinness for the express purpose of developing commercial products from scientific research.^66^ Before the patent was sealed in Great Britain, Richards and Hutchinson had submitted another application in the United States.^67^ Again, the patent related to nitrogenous fertilizer, but this expanded version added a fertilizer product along with the method of manufacture. The patent, granted in 1927, was assigned to ADCO.

ADCO was a British company. It was also the name of the trademarked product prepared by the patented process. Officially classed as an artificial reagent, the ADCO powder, when added to straw and wetted, effected the rapid decomposition of organic waste. The company advertised the end product as a synthetic farmyard manure and sold the powder internationally, with India being one of the places where ADCO was marketed.^68^

From its public reception, the ADCO starter and the Indore process were held in comparison. What ADCO offered was a composting alternative, minus the workload and bulk excrement.^69^ Though biological principles held consistent between locales, commentary on each method differed in economic terms. For example, a 1934 editorial on sanitation in India alluded to ADCO as a patented product that ‘hastens the formation of humus’, yet qualified its worth against the ‘pioneer work at Indore [which] has shown that the urine and dung of cows can adequately, and far more cheaply, replace the “Adco” mixture’.^70^ Paying for a powder to make compost hinted at absurdity in the context of rural India, a point similarly made by the Howards about chemical fertilizers. Price point favoured the Indore process.^71^

Clearly, employees at Rothamsted were not opposed to composting. They just viewed it as old and inexact.^72^ Much like scientists in the developing field of plant genetics, who sought to accelerate evolution by inducing mutations, soil chemists professed impatience with the time-tested ways of natural decomposition.^73^ The Indore process was too slow for those who believed that ‘quicker and better results’ were possible, and ADCO delivered ‘by adding to the heap some reagent to supply available nitrogen and lime’.^74^ The director of Rothamsted would later boast, ‘A certain precision has been conferred on the production of organic manures from waste materials that was quite absent from the older methods’.^75^

Advertisements for the patented product seem to cease after the 1930s. Nevertheless, the scientific research that resulted in the ADCO reagent produced other applications. Experiments at Rothamsted had demonstrated that ploughing straw directly into the land limited the nutrients available for plant growth. For the best results, they found that a ‘preliminary rotting of organic matter’ was needed to strike a sought-after ratio of carbohydrates and nitrogen.^76^ These recommendations, and the principles on which they rested, would later resurface in public critiques of Sir Albert Howard and the Indore process.

## Directors at odds

Historians have described the antagonism between Indore and Rothamsted as ideological in nature. The characterization would be more accurate by also identifying areas of overlap between the institutions and their respective personnel. For instance, employees at Rothamsted made a point to commend certain work done at the Indore Institute. ‘At Indore in India’, the head of the Chemistry Department for Rothamsted explained, ‘the ancient practice of composting plant refuse with soil, animal excreta, and lime was critically examined and standardized in the light of modern biochemical work’.^77^ In this way, Wad laid the basis for a positive scientific reception of the Indore process. Scientists valued his numerical analysis of composted material. Nonetheless, opposition to Indore and the Howards’ way of thinking about soil and plant health arose with respect to differing views about the consequences of agricultural research.

The nature of disagreement was displayed in 1937 after Sir Albert Howard gave a talk at the Royal Society of Arts in London. He introduced his paper as a follow-up to the proposal that agricultural policy should be based on soil fertility, summarizing his forty years of study from two perspectives, ‘the scientific and the practical’.^78^ Howard sided with farmers, who minded pragmatic results. Scientists in his experience had got things backwards. Of the various factors involved in the maintenance of fertility, the biological was foremost at Indore, then the physical, and lastly the chemical. Reversing this order – as was done by agricultural experiment stations like Rothamsted – produced grave results, in Howard’s opinion. To this point, he declared that ‘the use of artificial manures had failed’, substantiating his claim with reference to the mass death of earthworms caused by chemical top dressings.^79^

The ensuing discussion gravitated to the topic of earthworm necrosis. Professor R.G. Stapledon from Aberystwyth agreed with Howard’s thesis, yet challenged the idea that artificial manures destroyed earthworms. Stapledon swore that he could entice worms to follow him like an underground pied piper. The allusion was meant to illustrate the welcome earthworms felt on Welsh pastures broadcast with inorganic phosphates. This one concession aside, Stapledon agreed with Howard that practical improvements to soil fertility were readily available to ordinary farmers who understood decomposition.^80^ Other audience members were less agreeable, namely those connected with Rothamsted.

Given Howard’s dismissal of both chemistry and controlled field experiments, E.M. Crowther, an agricultural chemist at Rothamsted, saw little ground for debate in the paper. Its generic appeal to ‘success’ made scientific data immaterial. Still, Crowther refuted the idea that artificial fertilizers were ruinous to earthworms. Rothamsted had demonstrated a steady count of earthworms in plots under various treatments, among which were pastures fertilized with superphosphate for eighty years.^81^ The longitudinal evidence countered Howard’s grim imagery.

Although no longer employed at Rothamsted, Hutchinson was in attendance. He took a moment to question Howard’s self-citations. ‘In connection with some biological studies carried out at Rothamsted some years ago’, he reflected, ‘certain work was done which laid down broad lines for the production of artificial farmyard manure’; then, Hutchinson referenced a 1921 article written with fellow patentee Richards, where they had recommended a ‘more liberal use of litter in order to increase the amount of ammonia that can be fixed’.^82^ The scientific principles conveyed by these findings could readily explain the workings of ADCO or the Indore process. As a result, Howard’s portrayal of the originality of his work was somewhat misguided. Nothing in the lecture, it appeared to Richards, was new to science.

In the time remaining, Howard responded by reviewing the results of his lifelong education. ‘I have learned more from the diseases of plants and of animals than I have from all the professors of Cambridge, Rothamsted’ and the like, he declared.^83^ As far as earthworms were concerned, the numbers from controlled research plots were meaningless for deciding whether artificial fertilizer troubled soil biota. Howard was convinced that further studies would settle the matter in his defence: ‘I am going to rely on the one unanswerable argument – success – not on my own opinion or on other people’s opinion’.^84^ Howard gestured to his experiences, as did those from Rothamsted, and neither budged in their convictions.

Of all the chemists at Rothamsted, Sir Edward John Russell has been Howard’s most celebrated rival. Russell obtained a lectureship in chemistry at Wye Agricultural College in 1901, where he revised the syllabus to incorporate his own laboratory work that showed the improvement of plant growth in sterilized soils.^85^ Albert Howard joined Wye as a lecturer during Russell’s tenure there. This overlap is rarely mentioned, and its influence on both men can only be speculated. The truth is that Russell and Howard shared many interests.^86^ Both wanted to improve agriculture for the British Empire and the farming public. But how? Whatever professional hostility existed between the two men can be linked to their incompatible ideas about the primary directives of agricultural research. Then again, this ideological interpretation leaves open the question of institutional finances, a central consideration in setting scientific research agendas in the twentieth century.

Russell joined the Rothamsted Experimental Station as a soil chemist in 1907, accepting a post endowed by the Goldsmith’s Company, the bullion livery of London. He ascended to the directorship in the same year his book *Soil Conditions and Plant Growth* (1912) was published. The First World War soon changed the directives of the station. Fears of food scarcity prompted the British government to establish the Food Production Department. In doing so, ‘Rothamsted became the chief centre for dealing with soil and fertilizer problems and Russell was appointed technical adviser on this subject’, a colleague stated.^87^ Russell shifted his focus and, with Richards, began studying how to prolong the storage quality of manure. The scope of Russell’s expertise expanded over time to include, among other things, the topic of international agricultural development. By his retirement in 1943, Russell had become a well-known public figure.^88^

Russell visited India in the late 1930s. His notes from the trip – held by the Museum of English Rural Life – include the reflection that ‘in two centuries of domination the English did not succeed in alleviating the situation [of hunger], but in fact aggravated it’.^89^ The Rothamsted director believed that starvation in India dated to the Middle Ages, yet he felt that England had a responsibility to end it for good. Entire pages of struck-through text in his notebook from the trip remark on the topic of debt peonage, population growth and village life. What he witnessed led Russell to consider possibilities for the restoration of eroded land, a topic which ‘orthodox soil scientists and agricultural experts’ deemed somewhat heretical.^90^ Evidently, like Howard, he contemplated how to help ryots achieve a better life farming the land.

Russell read a paper to the Royal Society after returning from his tour of India. He observed that the per capita area of land devoted to food crops in India had declined since the First World War, with export commodity crops experiencing a steady rise. For Russell, the trend raised questions about what could be done to address hunger on the subcontinent. He identified village poverty and educational leadership as the primary causes of agrarian issues in rural India. From a quick survey, Russell had learned that few university graduates who studied agriculture entered the field as a profession. The director saw an opportunity for science to make a world of difference.^91^

Howard attended Russell’s talk and opened the discussion with a few comments about its overdose of statistics, finding it ‘quite impossible to regard them as a basis for serious argument’.^92^ Mixed cropping, fallow areas and rural food supplies went largely undocumented in the numbers Russell presented. Furthermore, as Howard illustrated through a focus on sugar cane, manuring tripled the yield of improved varieties in India: soil conditions could revolutionize agriculture for those following the Indore process. With this, the founder of the Indore Institute aired a concluding critique about the entire state of agricultural research. In Howard’s view, all the public money spent had ‘been wasted on isolated fragments’, repeatedly without ‘the slightest use to the cultivator’. The out-of-place approach to agricultural improvement and push for artificial fertilizers did nothing for people in India, according to Howard.^93^

After others’ remarks, the chairman for the Royal Society thanked the speaker and ended the forum by portraying village economic life in India as something to escape, through either higher crop prices or more produce per acre. In his mind, science was positioned to help with the latter. Evidence for this, he explained, could be found in the varieties developed by crop breeding programmes. Howard’s connection to this anecdote was likely not lost on Russell. Whereas a central concern of the Howards’ work at Indore was moving beyond scientific myopia to confront the economic reality of farming, agricultural chemists viewed science as their best available response for addressing rural poverty in India.

## Town waste royalties

Albert Howard became an active public speaker following his retirement in 1931. He used the platform on every occasion to promote the Indore process and, by association, his own reputation. Howard did not dispel the narrative that credited him solely for developing the Indore process (*sans* Wad, Gabrielle or ryots), although he did habitually redirect the ingenuity to nature itself. As its spokesperson, Howard glorified humus. His knowledge claims would be disputed by those with less-publicized composting methods backed by the legal protection of intellectual property rights.

After one public lecture in November 1933, a man in the London audience engaged Howard in a series of questions.^94^ The exchange with Mr Edward Henry Sams of Isleworth was captured by a stenographer: ‘Mr E.H. Sams said he was very interested in the subject as he had been making trials with house refuse and sewage sludge in this country since 1918’. Sams wanted to meet Howard again to discuss technical aspects of the Indore process, for he found the water content of British sewage to interfere with the aerobic process. Sams alleged that his own ‘company had overcome the difficulty of de-watering the sludge’ and making fertilizer from it ‘at less cost than the present method of burning house refuse, or dumping it’. Despite government apathy, the company already had three councils under contract, with more to sign soon.^95^

Howard took Sams up on his offer. In December 1934, they visited the Maidenhead Sewage Works to inspect Sams’s composting operation.^96^ ‘The process is patented’, Howard noted, and the ‘essence of the patent is the sorting and crushing’ that took place in making municipal compost. The Sams process first lifted town refuse – containing ‘coal & coke ashes, tin case, bottles’ – onto a grid, with waste paper crushed and arranged underneath to absorb liquid sludge. Howard was unimpressed with its partially decayed final product. In principle, he observed, bottles and cans would assist aeration if left in the ripening pile. ‘An obvious improvement’, Howard observed, ‘would be to spray the ordinary town refuse with sewage sludge and to mechanise the composting process from the beginning so as to promote more adequate aeration than is possible with the Sams process’.^97^ Howard wanted to experiment with these ideas. He chose to pitch a pilot project to the small municipalities of Ashford and Canterbury.

A year later, following a telephone conversation with Sams, Howard responded, ‘you very kindly suggested that I should join the Board of Directors of a company to develop your recent patent for converting municipal waste and sewage sludge into manure’.^98^ Howard explained that he ‘had decided not to accept any remuneration for my work in connection with the Indore process’ for two reasons. The first reason given was that Howard, as a pensioner, felt that ‘any practical results of my work are public property’.^99^ His stance echoed an older generation of peers in agricultural botany, who viewed their work as paradigmatic of a long-standing moral economy, repaid in kind, scientific credit and state funding.^100^ Howard had already received compensation for his work with the Indore process. Beyond pay, Howard’s career had earned him public acclaim for proselytizing about humus. This privilege was at stake in the proposal.

The second aspect of Howard’s stance, rejecting any business affiliation with Sams, had to do with the restrictive nature of patents. He professed that association with any patent would ‘seriously limit my freedom of thought and liberty of action in getting the Indore process taken up all over the world’.^101^ Whatever Howard might gain personally by joining a for-profit venture in municipal composting would come at the expense of his populist ambitions. He considered the proposed partnership with Sams a conflict of interest. Consistent with his sense of professional duty and the hard-won support of basic research, Howard justified his refusal through an ethos of public service. Lastly, Howard asserted that Sams’s patent ‘would not stand if the matter should come before the courts’ because of Howard’s own published works, as well the lecture where the two men had met.^102^

The content of this reply caught Sams by surprise. ‘You appear to me to be under some misapprehensions’, he wrote back. Sams clarified how the patents employed at Maidenhead dated back to 1920. For that matter, he had first learned how to compost as a youth apprentice in the market garden business. In Sams’s estimation, ‘the problem of treating sewage sludge for reducing its bulk has been with Civilised States since the introduction of the water carriage system’, but not until his own trials at a Kent sewage farm in 1918 was ‘a solution in sight’.^103^ There was no connection between his patent and the Indore process. If anything, the chronology would establish that priority was the other way around.

Sams denied having any previous knowledge of the Howards’ composting work in India, which entered print years after Sams had secured his patent. ‘I am of the opinion that your long absence in India has kept you out of touch with practical methods over here’, he lamented. Nonetheless, Sams encouraged Howard to develop a similar patent as his but in India. Great Britain was off limits. There was money in the mix. ‘I have some very influential financial interests supporting me’, Sams stated, with ‘some 30 to 40 Cities and Towns’ expected to implement the process. He ended the letter with a line reminiscent of Howard’s own philosophy: ‘Nature has been my teacher and master, not Man’.^104^

In the correspondence, Sams hinted at a number of patents filed under his name, the most recent being an application for securing cheap lime, a key ingredient in the composting process.^105^ His first patent for the fertilizer trade, filed in February 1920, had claimed a process for mixing pulverized house refuse with chalk.^106^ In August of that same year, Sams followed with a patent application which covered the basic process at Maidenhead, that of ‘feeding sewage sludge on to house refuse’.^107^ The patenting trend would continue. Two years after his exchange with Howard, Sams filed a patent to render peat usable for animal bedding or manuring, and he obtained rights to the same in the United States.^108^ His invention consisted of aerating a peat bed and sowing it with a green crop, which emitted heat during decomposition. The patented method mirrored practices generally used in composting. Dividing the process into constituent parts enabled Sams to obtain exclusive rights for a specific industrial application.

Not surprisingly, Howard wanted nothing to do with Sams’s company when visiting with the Cornwall County Council in October 1935 to discuss the utilization of municipal wastes. ‘All agreed not to get mixed up with patents’, he reported.^109^ Howard’s desire for Cornwall to take up municipal composting was such that he refused a salary if it were to happen.^110^ The doyen of compost viewed the current handling of waste by local authorities as uneconomical, but so was the Indore process in this case. ‘The cost of labour in this country is too great to permit the conversion of the municipal wastes and peat into humus by hand labour’, he wrote.^111^ To avert the expense, Howard proposed a mechanization of the Indore process and applied for a grant (£20,000) from the commissioner to get started. The situated knowledge from rural India was being resituated within the British industrial economy.

Howard’s plan for mechanizing the Indore process disclosed the impressions made from his visit to Maidenhead. He set out to improve on Sams’s design by hastening the rate of decomposition: ‘Unsorted town refuse and peat turves will be sprayed with sewage sludge as the mixed material passes through a rotary mixer’, inseminating the surface with fungi and bacteria, and reducing the time to thirty days. The method required no new machinery. In fact, Howard described the mechanized Indore method as ‘much simpler and cheaper’ than Sams’s process, and without royalties.^112^ The goal was to obtain humus for the benefit of the agricultural economy and its labour force. Howard declared, as if a foregone legal fact, that ‘it has been decided not to patent this method but to make it our personal contribution to the solution of the unemployment problem’.^113^ Whether or not this decision to refrain from filing for a patent represents a wholesale judgement against intellectual property, Howard avoided seeking exclusive rights in this instance with a self-fashioned magnanimity.

Howard’s commitment to public works was consistent with his larger civic vision. During his tenure in India, Howard developed an opinion that labourers needed basic amenities and the feeling of a ‘square deal’ to sustain productivity.^114^ This social theory accompanied his long-term thinking on state planning. In Howard’s world view, humus was a source of prosperity, and municipal composting provided the fertility needed for social reform. Howard outlined a land settlement scheme for England based on municipal humus, whereby ‘each acre of land will have to be supplied with a suitable dressing of lime and at least 50 tons of humus, free of cost to the smallholders, during the first two years, in order rapidly to raise the fertility to the proper stage of intensive production’.^115^ Ideally, this subsidy would encourage settlers to take an interest in soil welfare, a principle that he applied to whichever, or whosever, land was being settled.^116^

### Axiological sludge

The historical value of humus was a topic of intense debate among Howard’s contemporaries. In 1941, Lieutenant-Colonel F.C. Temple read a paper to the Royal Society of Arts about the manufacture of humus from municipal waste. Typical of these kinds of talk, Temple touched on the topic of public health and where things had gone wrong. ‘Almost every move in the improvement of agriculture was good and in the right direction’, he proclaimed, ‘until man tried his hand at the direct manufacture of fertiliser, instead of encouraging living organisms to do it for him’.^117^ To Temple, civic sanitation made waste less valuable by ushering it aside.^118^ One needed to look no farther than the river Thames for evidence.

Temple referred to humus as a mysterious thing and to Howard as someone with a solution to the problem of municipal waste. Strangely, Temple also stated, ‘The [Sams] process was publicly advocated by Mr. J.A. Coombs in 1936 and again by him and by Sir Albert Howard in 1938’.^119^ Adoption had been slow, according to the speaker, because it involved the coordination of two departments: sewage disposal belonged to London City Council and house refuse to borough councils.^120^ Plus, freight charges for the transport of dehydrated sludge cost the public money, with large quantities of filthy water spent in the process. Temple predicted that there should be ‘fertiliser factories at sewage works’ to divert these costs.^121^ Future compost sales factored into justifications for making the public investment.^122^

Audience members agreed that the disposal of sewage sludge was a practical problem needing to be addressed. On cue, a professor from Reading University voiced an objection. ‘I am a scientist’, he said, and ‘there is unfortunately creeping into this whole question a sort of mysticism which is suggestive of the Middle Ages rather than the twentieth century’.^123^ For the professor, the fact that healthy plants could be grown hydroponically, without any soil, exposed humus as being little more than a medium for water. He explained that the Agricultural Research Council had supported him with a grant to investigate compost along the lines at Maidenhead. From this research, he found it advisable to correct the ratio of carbon and nitrogen by adding chemical fertilizers to compost heaps.

The chairman of the Food Education Society followed by recalling how Sir Albert Howard had once fertilized a tract of land in India for three years and then stocked said land with cattle, none of which caught disease besides a known interaction with infected animals. The implication was that organic methods conferred exceptional health. The chairman submitted that a similar experiment should take place in Britain, as proposed in the *Medical Testament*, to compare the health of livestock and humans sustained by different production methods.^124^ A member of the Imperial Bureau of Soil Science in attendance called again for a needed clarity in terms. ‘Those who make humus a religion seldom specify what is meant by the term’, he remarked.^125^ In his version of history, the work done at Rothamsted had already defined the carbon–nitrogen ratio of decomposition. All that left for Howard was pile sorting and ambiguous vocabulary.

Another opinion emerged from the discussion. One attendee named Harold Sanderson regretted the difficulties faced by district councils in disposing of waste but disagreed with the terms of its current execution. ‘At Maidenhead an engineering firm has undertaken to do the work for the Council, at a price, but in my opinion that is not the right way’, said Sanderson. Councils, he believed, should establish industrial plants to manufacture their own humus. Sanderson exclaimed that England risked wasting a bountiful supply of fertility by outsourcing municipal composting.^126^ In his view, arriving at humus – whatever went into it – ought to be unmediated by the profits of private industry.

There was a consensus at the lecture that current disposal methods were unintelligent, but opinions diverged as to how to go about treating sewage and organic refuse. The various schemes under consideration were as much a matter of social solidarity as they were industrial engineering. Before closing the talk, Temple again invoked Howard, reiterating that ‘the value of compost must not be judged on comparison by chemical analysis with artificial fertilisers, but by results’.^127^ The Indore process, even if mechanized, stood as a testament to the potential benefits of dealing with collective waste.

## End of days

Howard spent his retirement promoting the life-giving properties of humus and opposing chemical reductionism. He was likely the outlier imagined when he predicted ‘the magic word Science will be freely employed to bludgeon the iconoclast’.^128^ This depiction of an embattled fate helped the organic movement to craft its narrative for an alternative agriculture. Thoughout his life, Howard used his growing publicity to challenge the provisional success of chemical fertilizers. His concerns gravitated towards the interests of farmers making a living on the land, rather than credibility among scientific peers.

Of all people, Russell was asked to write Howard’s obituary in 1947. ‘No new principle was involved in the work’ at Indore, he eulogized. Employees at Rothamsted could point to their own publications to discredit the idea that Howard had introduced a new scientific understanding of decomposition. For Russell, the Howards’ ideas were redeemed by having ‘caused scientific workers in agriculture to re-examine the foundations on which their hypotheses are based – always a useful exercise’.^129^

This statement by Russell offers insight into the scientific contests around soil fertility in the first half of the twentieth century. The study of agriculture had fractured into isolated fields of expertise, each with their own firmly held facts. The Howards wanted scientists to re-examine the usefulness of *that* exercise. What good were academic minutiae and statistical models to those trying to farm for a living? The Howards came to what they believed was a more basic truth. Humus helped farmers. As a result, the Indore process was freely copied all over the world.^130^

Widespread affordability was a major concern at the Indore Institute. True to the early organic ethos, Howard and Wad once wrote that the Indore process could transform any cultivator into ‘a chemical manufacturer’.^131^ Agriculture stood to prosper through the application of science so long as technical findings were made practical for impoverished rural households with limited income. The modernizing project of ‘cultivator self-help’ in India, however, was transformed by interwar economic forces and capitalist development discourse.^132^ When Howard returned home, he had looked to mechanize the Indore process for the English labour market, now with town waste as a precursor to much-needed humus formation.

Questions about who would provision soil fertility and how they should be remunerated played out in negotiations involving patents. Howard shied away from patents as a pensioner, for he felt that taxes and levies had already covered the expenses of his public research. Rothamsted employees adopted a different approach with ADCO and assigned patent rights to a non-profit commercial entity. They repaid Guinness’s patronage in this way. Sams, for his part, sought to recoup private investment and turn a profit with his intellectual property. He did so by targeting contracts with public works. Research funding had an influence on what was done with research findings.

These cases cannot be glossed in categorical opposition based on material terms alone. Agricultural chemistry and engineering generated know-how about decomposition that could be specified for patent protection, whereas a holistic treatment of humus in village economic life was less susceptible to the same monopolistic rights. Ultimately, Sir Albert Howard attained an authority beyond science for the practical lessons learned while studying soil fertility. His rivals, sceptical at best, countered the novelty of the Indore process with evidence of their own, including legal title to intellectual property. As this article has shown, property claims were a key factor in making knowledge about compost during the first half of the twentieth century.

## Footnotes

1 Gregory A. Barton, ‘The myth of the peasant in the global organic farming movement’, *Itinerario* (2017) 41(1), pp. 75–91; J. Heckman, ‘A history of organic farming: transitions from Sir Albert Howard’s *War in the Soil* to USDA National Organic Program’, *Renewable Agriculture and Food Systems* (2006) 21(3), pp. 143–50; David R. Hershey, ‘Sir Albert Howard and the Indore process’, *HortTechnology* (1992) 2(2), pp. 267–9. For another perspective see John Paull, ‘Lord Northbourne, the man who invented organic farming, a biography’, *Journal of Organic Systems* (2014) 9(1), pp. 31–53.

2 Albert Howard, *An Agricultural Testament*, London: Oxford University Press, 1940.

3 A. Howard, op. cit. (2), pp. 22–3; Greta Marchesi, ‘Justus von Liebig makes the world: soil properties and social change in the nineteenth century’, *Environmental Humanities* (2020) 12(1), pp. 205–26. For a prehistory see Peter M. Jones, ‘Making chemistry the “science” of agriculture, c. 1760–1840’, *History of Science* (2016) 54(2), pp. 169–94.

4 Thomas F. Gieryn, *Cultural Boundaries of Science: Credibility on the Line*, Chicago:University of Chicago Press, 1999, p. 286.

5 Daniel Münster, ‘Fermentation, soil health, and bionativism in Indian natural farming’, *Current Anthropology* (2023) 62(24), pp. S311–S322; Shaila Seshia Galvin, *Becoming Organic: Nature and Agriculture in the Indian Himalaya*, New Haven, CT: Yale University Press, 2021; Vandana Shiva, ‘Earth democracy: sustainability, justice, and peace’, *Buffalo Environmental Law Journal* (2019) 26(1), pp. 1–15.

6 Jonathan Liebenau, ‘Paul Ehrlich as acommercial scientist and research administrator’, *Medical History* (1990) 34, pp. 65–78; Jack Ralph Kloppenburg, *First the Seed: The Political Economy of Plant Biotechnology*, Madison: University of Wisconsin Press, 2005; Mark R. Finlay, ‘Science, promotion, and scandal: soil bacteriology, legume inoculation, and the American campaign for soil improvement in the Progressive Era’, in D. Phillips and S. Kingsland (eds.), *New Perspectives on the History of Life Sciences and Agriculture*, Cham: Springer International Publishing, 2015, pp. 205–29; Jenny Bulstrode, ‘The industrial archaeology of deep time’, *BJHS* (2016) 49(1), pp. 1–25; Joseph D. Martin, ‘Patronage of basic research at the University of Chicago, 1945–1953’, *Isis* (2020) 111(4), pp. 697–716.

7 Philip Conford, ‘The myth of neglect: response to the early organic movement, 1930–1950’, *Agricultural History Review* (2002) 50(1), pp. 89–106.

8 Conford, op. cit. (7), p. 106.

9 Thomas Parke Hughes, ‘Technological momentum in history: hydrogenation in Germany 1898–1933’, *Past & Present* (1969) 1(44), pp. 106–32. For other examples see Adam M. Romero, *Economic Poisoning: Industrial Waste and the Chemicalization of American Agriculture*, Oakland: University of California Press, 2022.

10 Barton, op. cit. (1), p. 75.

11 F.H. King, *Farmers of Forty Centuries, or Permanent Agriculture in China, Korea, and Japan*, Oxford: Benediction Classics, 2011 (first published 1911).

12 Gieryn, op. cit. (4), p. 300.

13 A. Howard, op. cit. (2), p. ix.

14 Merlin Sheldrake, ‘Albert Howard and the mycorrhizal association’, *Studies in History and Philosophy of Biological and Biomedical Sciences* (2012) 43, pp. 225–31, 230.

15 Sanjukta Ghosh, ‘Scientific knowledge and practices of green manuring in Bengal Presidency, 1905–1925’, in E. Rashkow, S. Ghosh and U. Chakrabarti (eds.), *Memory, Identity and the Colonial Encounter in India: Essays in Honour of Peter Robb*, London: Routledge India, 2017, pp. 298–319.

16 Conford, op. cit. (7), p. 91.

17 Gieryn, op. cit. (4), p. 305.

18 Gieryn, op. cit. (4), pp. 310, 323.

19 University of Cambridge, St John’s College Library, Papers of Sir Albert Howard (subsequently HowardA), A. Moloney to A. Howard, 3 September 1900, Folder A/6/1.

20 E. John Russell, ‘Sir Albert Howard, C.I.E.’, *Nature* 4074 (29 November 1947), pp. 741–2.

21 Louise E. Howard, *Sir Albert Howard in India*, London: Faber and Faber, 1953, p. 5 (pagination from digital version).

22 L. Howard, op. cit. (21), p. 18, quoting a letter from G. Howard of 28 July 1905.

23 G. Howard to her mother, 21 April 1905, HowardA, Folder B/1/1.

24 L. Howard, op. cit. (21), p. 6.

25 L. Howard, op. cit. (21), p. 6.

26 L. Howard, op. cit. (21), pp. 39–44.

27 HowardA, Folder B/1/4.

28 G. Howard to her mother, 19 September 1910, HowardA, Folder B/1/5.

29 Albert Howard and Gabrielle L.C. Howard, *The Development of Indian Agriculture*, Bombay: Humphrey Milford, Oxford University Press, 1927, p. 7.

30 L. Howard, op. cit. (21), p. 17.

31 For a legal history of ginning, fibre and cotton varieties in India see Kencho Peldon, ‘Last, the plant: governing cotton in British Colonial India, 1850–1942’ (PhD thesis, University of Queensland, 2024).

32 Gieryn, op. cit. (4), p. 293; F.L. Engledow, ‘The application of science to crop production: an experiment carried out at the Institute of Plant Industry, Indore’, *Nature* (1929) 124, pp. 974–6.

33 Thomas F. Gieryn, ‘Three truth-spots’, *Journal of the History of the Behavioral Sciences* (2002) 38(2), pp. 113–32; Ghosh, op. cit. (15), p. 305.

34 Russell, op. cit. (20), p. 741.

35 Albert Howard and Yeshwant D. Wad, *The Waste Products of Agriculture: Their Utilization as Humus*, London: Humphrey Milford, Oxford University Press, 1931, p. 60; *Institute of Plant Industry, Indore: Press Notices on the First Quinquennial Review of the Work of the Institute*, Calcutta: Baptist Mission Press, HowardA, Folder A/1/2.

36 Ghosh, op. cit. (15), p. 300.

37 Albert Howard, ‘The manufacture of humus by the Indore process’, *Journal of the Royal Society of Arts* (1935) 84(4331), pp. 26–59.

38 Howard and Wad, op. cit. (35).

39 Yeshwant D. Wad, ‘The work at Indore’, *Organic Gardening* (1948) 13(3), pp. 6–9.

40 *Annual Report of the Indian Central Cotton Committee, Bombay, for the Year Ending 31st August 1928*, Bombay: G. Claridge & Co., Ltd, 1928, p. 117.

41 ‘Disease resistance in plants’, *Nature* (1937) 139(3518), p. 598; Sir John Russell, *Report on the Work of the Imperial Council of Agricultural Research in Applying Science to Crop Production in India*, Delhi: Government of India Press, 1937, p. 176.

42 On this history see Gregory T. Cushman, *Guano and the Opening of the Pacific World: A Global Ecological History*, Cambridge: Cambridge University Press, 2013.

43 L. Howard, op. cit. (21).

44 Mentioned in John Paull, ‘*The Global History of Organic Farming*, by Gregory A. Barton’, *English Historical Review* (2019) 134(570), pp. 1346–8.

45 *Annual Report of the Indian Central Cotton Committee, Bombay, for the Year Ending 31st August 1931*, Bombay: G. Claridge & Co., Ltd, 1932, Appendix A, p. 80.

46 Wad, op. cit. (39), p. 9.

47 Wad, op. cit. (39), p. 7.

48 Consider this addendum by the chairman after text of Howard’s talk: ‘At present we know nothing of humus, beyond that it is good food for plants – the best they can have … As to its nature, the question is, whether it be not largely a vital product, not a mere organic residue.’ A. Howard, op. cit. (37), p. 58.

49 The Rothamsted Experimental Station was never a public institution, but it did, at times, receive funding through government grants.

50 Prior to the work of Liebig, ‘artificial manure’ referred to a fertilizer made of marl or bones, yet after the 1840s the term also denoted a chemical product of industrial manufacture. See Jones, op. cit. (3), p. 184 n. 62.

51 Richard P. Aulie, ‘The mineral theory’, *Agricultural History* (1974) 48(3), pp. 369–82.

52 Patent no. GB 9,353, applied on 23 May 1842 (granted November 1842).

53 E. John Russell, ‘Rothamsted and its experiment station’, *Agricultural History* (1942) 16(4), pp. 161–83, 171.

54 Museum of English Rural Life, University of Reading (subsequently MERL), TR LAW SP1/23/2.

55 Quoted in Russell, op. cit. (53), p. 173.

56 Russell, op. cit. (53); *Lawes*’ *Patent Chemical Manures, Twenty-Fourth Circular, Spring, 1864*, MERL, TR LAW P2/A1.

57 Lawes’ Chemical Manure Co. booklet, n.d., MERL, TR LAW P2/A6, p. 4.

58 Ian Angus, ‘Cesspools, sewage, and social murder: environmental crisis and metabolic rift in nineteenth-century London’, *Monthly Review* (2018) 70(3), pp. 32–68, 34.

59 Karen Sayer, ‘At the farm gate: decomposition, decay and destruction in field and farm in Victorian Britain’, in A. Alyal, S. Anderson and R. Mitchell (eds.), *Victorian Cultures of Liminality: Borders and Margins*, Newcastle upon Tyne: Cambridge Scholars Publishing, 2018, pp. 107–36.

60 W. Harwood Long, ‘The development of mechanization in English farming’, *Agricultural History Review* (1963) 11(1), pp. 15–26.

61 E.J. Russell, ‘Mr E H Richards’, *Nature* (1939) 143(3632), pp. 968–9.

62 E.H. Richards, ‘The fixation of nitrogen in faeces’, *Journal of Agricultural Science* (1917) 8(3), pp. 299–311; H.B. Hutchinson, ‘The influence of plant residues on nitrogen fixation and on losses of nitrate’, *Journal of Agricultural Science* (1918) 9(1), pp. 92–111; H.B. Hutchinson and James Clayton, ‘On the decomposition of cellulose by an aerobic organism (*Spirochaeta Cytophaga, N. Sp*.*)*’, *Journal of Agricultural Science* (1919) 9(2), pp. 143–72.

63 Patent no. US 1,471,979, filed on 12 August 1921 (granted 23 October 1923).

64 Patent no. US 1,471,979, pp. 8–9.

65 Patent no. GB 219,384, filed on 23 April 1923 (granted 16 October 1924).

66 *Report 1921*–*22 with the Supplement to the* ‘*Guide to the Experimental Plots*’ *Containing The Yields per Acre etc*., Harpenden: Rothamsted Research (1923), p. 20; H.D. Kay, ‘Rupert Edward Cecil Lee Guinness, Second Earl of Iveagh, 1847–1967’, *Biographical Memoirs of Fellows of the Royal Society* (1968) 14, pp. 287–307; Russell, op. cit. (61), p. 968.

67 Patent no. US 1,619,679, filed on 15 April 1924 (granted 1 March 1927).

68 Advertisement in V.E. Wilkins, *Research and the Land: An Account of Recent Progress in Agricultural and Horticultural Science*, London: His Majesty’s Stationery Office, 1927, p. viii.

69 Sir E.J. Russell, ‘The effect of mechanization on soil fertility’, in *Rothamsted Conferences XIV, Mechanization and British Agriculture*, Harpenden: Rothamsted Experimental Station, 1933, pp. 7–16; E.M. Crowther, ‘Agricultural chemistry, 1910–1935’, *Chemistry and Industry* (1935) 54(18), pp. 422–5.

70 A.D.S., ‘Waste, wealth, and health’, *Indian Medical Gazette*, February 1934, 85–8, 86.

71 Howard tried ADCO in India, and he ‘found it did not work so well as the Indore method’. Actually, he was convinced that it was ‘bound to fail’ because ‘no permanent or effective system of agriculture will ever be devised without the use of the waste products of the animal’. A. Howard, op. cit. (37), p. 55.

72 H.V. Garner, ‘Composts’, *Journal of the Ministry of Agriculture* (1937–8) 44(4), pp. 330–4, 330.

73 See Helen Anne Curry, *Evolution Made to Order: Plant Breeding and Technological Innovation in Twentieth-Century America*, Chicago: University of Chicago Press, 2016.

74 Garner, op. cit. (72), p. 332.

75 Russell, op. cit. (69), p. 11.

76 Russell, op. cit. (69), p. 11.

77 Crowther, op. cit. (69), p. 424.

78 Sir Albert Howard, ‘The restoration and maintenance of fertility’, *Journal of the Farmers*’ *Club*, Part 1, February 1937, pp. 1–18, 2.

79 A. Howard, op. cit. (78), p. 3.

80 A. Howard, op. cit. (78), pp. 8–9.

81 A. Howard, op. cit. (78), pp. 11–12.

82 A. Howard, op. cit. (78), pp. 15–16.

83 A. Howard, op. cit. (78), p. 17.

84 A. Howard, op. cit. (78), p. 18.

85 H. Gerard Thornton, ‘Edward John Russell. 1872–1965’, *Biographical Memoirs of Fellows of the Royal Society* (1966) 12, pp. 456–77, 461.

86 E.J. Russell, ‘Science and the Indian peasant’, *Journal of the Royal Society of Arts* (1939) 87(4512), pp. 662–81.

87 Thornton, op. cit. (85), p. 463.

88 Firman E. Bear, ‘Sir John Russell’, *Plant Physiology* (1944) 19(3), pp. 391–3, 393.

89 MERL, FR HERT 11/5/18, p. 1.

90 MERL, FR HERT 11/5/18, pp. 4–6.

91 Russell, op. cit. (86).

92 Russell, op. cit. (86), p. 675.

93 Russell, op. cit. (86), p. 676.

94 A. Howard, ‘The waste products of agriculture: their utilization as humus’, *Journal of the Royal Society of Arts* (1933) 82(4229), pp. 84–121, HowardA, Folder A/1/2.

95 A. Howard, op. cit. (94).

96 *Notes on the Sams Process (Sewage Study and Town Refuse)*, 18 November 1934, HowardA, Folder A/1/2.

97 *Notes on the Sams Process*, op. cit. (96).

98 A. Howard to E.H. Sams, 24 September 1935, HowardA, Folder A/1/2.

99 Howard to Sams, op. cit. (98).

100 Berris Charnley, ‘Seeds without patents: science and morality in British plant breeding in the long nineteenth-century’, *Revue économique* (2013) 64(1), pp. 69–88; Christine MacLeod, ‘Reluctant entrepreneurs’, *Isis* (2012) 103(2), pp. 328–39; see also Xan Sarah Chacko, ‘When life gives you lemons: Frank Meyer, authority, and credit in early twentieth-century plant hunting’, *History of Science* (2018) 56(4), pp. 432–69.

101 Howard to Sams, op. cit. (98).

102 Howard to Sams, op. cit. (98).

103 Howard to Sams, op. cit. (98).

104 Howard to Sams, op. cit. (98).

105 Patent no. GB 451,885A, filed on 29 May 1935 (granted 13 August 1936).

106 Patent no. GB 161,777, filed on 17 February 1920 (granted 21 April 1921)

107 Patent no. GB 163,417, filed on 30 August 1920 (granted 26 May 1921).

108 Patent no. US 2,178,051, filed on 16 May 1938 (granted 13 October 1939).

109 A. Howard, *Notes on a Visit to Cornwall*, 1935, HowardA, Folder A/1/2.

110 Compare this philanthropic stance with that of Eleanor Ormerod, who received no salary from the Royal Agricultural Society of England for her entomological work. J.F.M. Clark, ‘Ormerod, Eleanor Anne’, *Oxford Dictionary of National Biography*, at www.oxforddnb.com/view/10.1093/ref:odnb/9780198614128.001.0001/odnb-9780198614128-e-35329 (accessed 1 December 2023).

111 Albert Howard, *Unemployment and the Land: A Practical Contribution to the Problem* (August 1935), HowardA, Folder A/1/2/, p. 3.

112 A. Howard, op. cit. (111), pp. 3–4.

113 A. Howard, op. cit. (111), p. 4.

114 Albert Howard, ‘An experiment in the management of Indian labour’, *International Labour Review* (1931) 23(5), pp. 636–43, 641.

115 A. Howard, op. cit. (111), p. 5.

116 Albert Howard took an independent contracting job in 1935 to report on a land development project in Morocco called Tafrata Estates, a 75,000-hectare concession ceded by the sultan to a Parisian investor. Albert Howard, ‘The development of Tafrata Estates’, HowardA, Folder A/1/2. On the history of Tafrata Estates see Adam Guerin, ‘Deserts, capital and “civilisation”: the politics of environmental naming in eastern Morocco, 1925–1939’, *Global Environment* (2019) 12(1), pp. 134–53.

117 F.C. Temple, ‘Municipal manufacture of humus from habitation wastes’, *Journal of the Royal Society of Arts* (1941) 89(4582), pp. 215–28, 216.

118 Temple, op. cit. (117), p. 217.

119 Temple, op. cit. (117), p. 220. I did not find where Howard publicly advocated for the Sams process.

120 Chris Otter claimed that ‘such projects were inconsequential’ in the postwar period, as chemical fertilizers eutrophied British waterways. Chris Otter, *Diet for a Large Planet: Industrial Britain, Food Systems, and World Ecology*, Chicago: University of Chicago Press, 2020, p. 229.

121 Temple, op. cit. (117), pp. 221–2.

122 Daniel Schneider, *Hybrid Nature: Sewage Treatment and the Contradictions of the Industrial Ecosystem*, Cambridge, MA: MIT Press, 2011.

123 Temple, op. cit. (117), p. 224.

124 On the *Medical Testament* as a key text linking agricultural practices and human health see Laura Sayre, ‘Food, farming and universal health: the *Medical Testament* and the organic movement’, *Global Environment* (2018) 11(1), pp. 130–53; Conford, op. cit. (7), pp. 99–100.

125 Temple, op. cit. (117), p. 226.

126 Temple, op. cit. (117), p. 225.

127 Temple, op. cit. (117), p. 221.

128 Quoted in Gieryn, op. cit. (4), p. 315.

129 Russell, op. cit. (20), p. 742.

130 Examples of the Indore process from Kenya and Ceylon appear in A. Howard, op. cit. (37).

131 Howard and Wad, op. cit. (35), p. viii.

132 Prakash Kumar, ‘“Modernization” and agrarian development in India, 1912–52’, *Journal of Asian Studies* (2020) 79(3), pp. 633–58.

